# The impact of HIV and AIDS research: a case study from Swaziland

**DOI:** 10.1186/1478-4505-9-S1-S9

**Published:** 2011-06-16

**Authors:** Alan Whiteside, Fiona E Henry

**Affiliations:** 1 Director and Professor, Health Economics and HIV/AIDS Research Division University of KwaZulu-Natal, Durban; 2 Visiting Fellow, Health Economics and HIV/AIDS Research Division University of KwaZulu Natal, Durban

## Abstract

**Background:**

Swaziland is experiencing the world’s worst HIV and AIDS epidemic. Prevalence rose from four percent of antenatal clinic attendees in 1992 to 42.6 percent in 2004. The Report ‘Reviewing ‘Emergencies’ for Swaziland: Shifting the Paradigm in a New Era’ published in 2007 bought together social and economic indicators. It built a picture of the epidemic as a humanitarian emergency, requiring urgent action from international organisations, donors, and governments. Following a targeted communications effort, the report was believed to have raised the profile of the issue and Swaziland - a success story for HIV and AIDS research.

**Methods:**

Keen to understand how, where and why the report had an impact, Health Economics and HIV/AIDS Research Division commissioned an assessment to track and evaluate the influence of the research. This tapped into literature on the significance of understanding the research-to-policy interface. This paper outlines the report and its impact. It explores key findings from the assessment and suggests lessons for future research projects.

**Results:**

The paper demonstrates that, although complex, and not without methodological issues, impact assessment of research can be of real value to researchers in understanding the research-to-policy interface.

**Conclusion:**

Only by gaining insight into this process can researchers move forward in delivering effective research.

## Background

### HIV and AIDS Research in Swaziland

#### The HIV and AIDS Epidemic

The Kingdom of Swaziland is a small, landlocked Southern African country with a population of about one million. In the 1980’s it experienced a significant upturn in investment and growth, benefiting from a regional advantage due to war in Mozambique and apartheid in South Africa. This was combined with relative political stability, sound macroeconomic policies, and a cheap and productive workforce, and resulted in significant inflows of foreign direct investment. Between 1985 and 1999 the growth rate was 6% and the well-being of the Swazi’s was on a modest upward trajectory [1]. Since 2000 these gains have been reversed, largely due to the impact of HIV and AIDS.

Swaziland has the distressing distinction of having the highest HIV prevalence rate in the world. Its epidemic spread with exceptional speed, from four percent of antenatal clinic attendees in 1992 to 42.6 percent in 2004 [2]. The 2006 data seemed to offer some hope with a decline to 39.2 percent, but the 2008 survey recorded a rise to 42 percent [3]. Mortality rose and life expectancy fell from 60 years in 1997 to 31.3 years in 2004, the world’s lowest [4]. Swaziland is additionally handicapped because the economic success of the 1980s and early 1990s mean it is categorised as a middle-income country, and cannot access international support available to low-income countries.

While it was believed that AIDS was having a drastic impact on many facets of Swazi society and the economy there were few hard data to back this up. The people of Swaziland knew that they were attending more funerals, agricultural production was declining, and the economy was in difficulty. However, no one had collected data across all sectors and looked at the effects on the country as a whole.

In early 2007 the terms of reference for a study to assess the impact of the AIDS epidemic across the nation were drawn up. The ideas were developed primarily by Dr Derek Von Wissell, Head of the National Emergency Response Council on HIV/AIDS in Swaziland (NERCHA) and Professor Alan Whiteside, Director of the Health Economics and HIV/AIDS Research Division (HEARD) at the University of KwaZulu-Natal. The research was partly funded by HEARD and led by Amy Whalley, a former Overseas Development Institute Fellow who had worked with NERCHA and the Ministry of Health and guided by Whiteside and staff at NERCHA. Whalley’s task was to gather and analyse information on what was going on across the nation using available data sets; to compare Swaziland’s situation with other countries and thus build advocacy material for use inside the country and with the international community. The write up was primarily by Whalley with major input by Whiteside. In October 2007 the report, ‘Reviewing ‘Emergencies’ for Swaziland: Shifting the Paradigm in a New Era’, (here after called ‘Reviewing Emergencies’) was published [5], distributed and disseminated. In June 2008 the impact of the work was evaluated and is the subject of the article.

#### Reviewing Emergencies Report

Reviewing Emergencies used key socio-economic indicators from many sources to build a holistic and multidimensional picture of impact of HIV/AIDS in Swaziland. Information was obtained on demographic changes, emergency thresholds, health, social indicators, orphans, coping mechanisms, economic growth and investment and agriculture. These were tracked over time. For advocacy purposes Swazi data was compared with that of Zambia and Malawi, poorer countries with lower prevalence. The report painted a bleak picture showing that Swaziland is experiencing a humanitarian crisis comparable to countries besieged by conflict or struggling in the wake of a severe natural disaster. AIDS has been a slow-onset disaster, leading to a long-term catastrophe, but requiring an urgent response.

**Figure 1:**
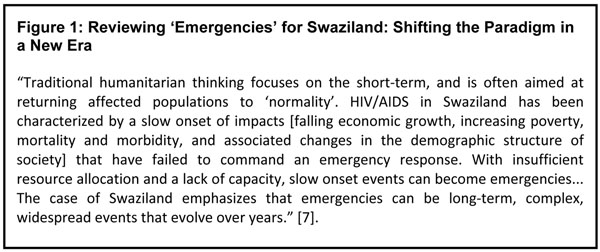
Reviewing ‘Emergencies’ for Swaziland: Shifting the Paradigm in a New Era

The report spoke to two audiences. For Swazi’s it confirmed that AIDS was indeed having a devastating effect on their nation. The same message was aimed at the international community. However it also urged the latter to re-examine the HIV and AIDS epidemic. It sought to broaden the traditional consensus on what constitutes an emergency to include ‘long-wave emergencies’. Effective interventions require both an immediate emergency response but also have to build capacity for long-term programmes [5]. In the course of the research the country’s classification emerged as an important issue. As a lower-middle income country Swaziland is not eligible for international development assistance (IDA) grants from the World Bank, and concessional-lending [6]. The report challenged the use of GDP per capita as an indicator to set the status of a country and its access to support in the face of a generalised AIDS epidemic. It noted the global perception of ‘middle-income countries’ is that they need less support and are somehow ‘less deserving’. (Figure 1)

## Methods

### Assessing the impact: The research to policy interface

Much of SRH and HIV and AIDS research, particularly in the development arena, aims to influence policy, it is “research committed to improvement” [7]. Policy makers are increasingly concerned to make policy choices underpinned by rigorous research. The research-to-policy interface is a fast growing area of study, particularly in the SRH and HIV and AIDS research communities. The purposes are two fold: accountability of a research organisation, demonstrating achievement and value for money for funders; and as a learning exercise, developing a better understanding of the research impact process in order to enhance future impact [8]. HEARD is an applied research organisation aiming to mobilise evidence for interventions in health and HIV in the region [9]. Only by identifying where change has transpired as a result of its research, where it has not, and the reasons for this, can it deliver effective research.

Impact assessment is an underdeveloped field of study, in part due to the complex and dynamic nature of research impacts and the consequent difficulty in measuring them. Sumner, Perkins and Lindstrom (2008, unpublished) identify a number of significant problems when attempting to track the impact of research: difficulty in determining conceptual influence (on opinion, attitudes and thinking); identifying research users, timing of assessment; attributing impact in the context of other drivers; and using qualitative and subjective data [10]. Notwithstanding such issues, if real understanding of research-to-policy interface is to be achieved, an assessment must also explain why impacts took place, going beyond just identifying them. Difficult as it may be, there are good reasons for attempting to evaluate the impact of policy research [11]. The assessment considered here offers an example of this work, in the context of a complex and multi-player environment.

#### Methodology and issues

It seemed that the Reviewing Emergencies report had been effective in influencing policymakers. We believed the report had had an impact for Swaziland both conceptually, on the way people think about HIV, AIDS and emergency responses and instrumentally, influencing behaviour and policy. In mid 2008, the decision was taken to carry out an assessment of the impact of the report to determine the validity of this claim and understand what ‘worked’ and what ‘didn’t’. Fiona Henry, who was awarded a fellowship by the University of Edinburgh to work with HEARD, was tasked with leading the assessment.

The specific objectives were to:

• Document the creation and dissemination of the report;

• Identify and explain its impact;

• Identify any barriers and/or limitations to its impact;

• Draw lessons for maximising the impact of future research.

This article was developed from the assessment assisted by a presentation given at the meeting of DFID funded Research Programme Consortia on ‘Strengthening the research to policy and practice interface: Exploring strategies used by research organisations working on Sexual and Reproductive Health and HIV and AIDS’ held in Liverpool in May 2009; and through peer review. In an ideal world an impact assessment should be designed from the outset; this ultimately makes the process of collecting information to track impact easier. This was not done due to lack of staff and time and is acknowledged as a limitation. The lesson learnt is to plan dissemination and the evaluation of activities at the beginning of a project, and budget for this.

#### Forward-tracking and attribution

Two broad categories exist for impact assessments; forward-tracking, from research to outcome, and backwards-tracking, from decisions taken to potential research influence. Our impact assessment wanted to track from publication to outcome. However, forward-tracking approaches can have serious limitations [12]. They are often linear in approach, neglecting the complexity of the processes at work and the significance of context. The policy environment is influenced by socio-cultural, political and economic factors and these must be acknowledged in order to understand why an impact took place. Taking this into account, the assessment attempts to put identified ‘impacts’ into a relevant context.

The assessment cannot claim to fully understand the influence of other ‘drivers’ on outcomes. Policy research is only one of many sources of information used in decision making or to form opinion. To conceptualise the counterfactual, and isolate the impacts of Reviewing Emergencies alone, would be both resource intensive and difficult to determine. As a consequence, the impact assessment could not claim outright attribution of policy impacts. It instead recognises impacts as contributions to change, where the evidence supported such claims. This difficult methodological issue of attributing outcomes can result in a ‘shying away’ from impact assessments [10]. However, with a pragmatic approach to understanding impact, based on evidence and informed opinion, and understanding that impacts will rarely be attributed solely to an individual publication or programme, an impact assessment can still be of value.

#### Conceptualising ‘impact’: A temporal approach

‘Impact’ is used interchangeably with terms such as ‘influence’, ‘outcomes’, ‘use’ and ‘uptake’, and a number of definitions exist in the literature [10]. In the assessment of the Reviewing Emergencies impact is defined temporally, referring to ‘initial impact’, ‘long-term impact’ and ‘potential impact’. This is important. Firstly, initial impact refers to the ‘sticky messages’ of the report: what strikes the reader instantly about the report and its findings and the key messages that they come away with. Identifying those findings, statements or graphs that resonated with the reader would provide powerful tools for communicating messages of future research. Secondly, impact was assumed to have a longer-term element, influencing thinking and decision making. This constituted the main body of the assessment. ‘Long-term impacts’ are those conceptual and instrumental impacts that change understanding and attitudes or contribute to a change in policy or behaviour. As the assessment took place about a year after the launch of Reviewing Emergencies, ‘potential impact’ considered the possibility of impact in the future. With continued advocacy, and changes to the policy environment, potential impact outlines the ‘capability’ of the report’s findings. It highlights areas in which to focus advocacy efforts in the future.

#### Research users

Policy research can be used for multiple, often unforeseen, purposes [11]. Tracking a research contribution, especially one that seeks conceptual change, is difficult. Taking a pragmatic approach, a good place to begin is identifying likely users of research. The impact assessment chose five sectors for analysis to try to encompass key actors. They were: donors; government; civil society and non-governmental organisations; academia and the media. Identifying them helped to structure the analysis and understand the different ‘uptake’ of the research. The categories were purposefully broad in recognition of the broad array of policy players, and to enable flexibility in analysis; crossing national boundaries and disciplines. Lessons from Swaziland, we believed, would be applicable elsewhere in the region, especially in Lesotho, Namibia and Botswana as these are all defined as lower-middle-income countries; have similar prevalence levels; and are members (with South Africa) of the Southern African Customs Union. It was also important as Ministries of Health are often weak and in many African countries donor policies have a disproportionate influence on health.

#### Data and measurement

‘Measuring’ impact posed some difficulty. Changes to thinking and decisions are particularly hard to quantify. For this reason a qualitative approach was used, asking how and why people believed the report had altered their approach to the Swazi epidemic, and what impact they believed the report had. Anecdotal evidence and substantive examples were key to supporting such beliefs in the absence of quantitative evidence. Impact was ultimately considered against the aims of the report: determining what was achieved as intended, what was not achieved, and any unintended impacts.

The methods consisted first of a literature review, to develop understanding of the background and terms of reference for the study. Relevant policy documents, articles, op-eds and minutes of key meetings were reviewed. A questionnaire with questions relating to influence to date, potential influence and barriers to influence across sectors was distributed to 50 individuals, in the five sectors. Questions asked the respondents to rank how influential they thought the report had been in different areas, from ‘no influence’ to a ‘very large influence’ (including a ‘don’t know’ option). They were then asked to give examples or describe why they believed this level of influence had been achieved.

Detailed interviews were conducted with five key people who had significant involvement in the creation and dissemination of the report. Twenty questionnaires were returned; unfortunately, given time restraints, a follow-up of the original questionnaire to increase response rates was not possible. In analysing feedback from questionnaires, the percentage of answers for each ranking were calculated. Similar details or examples from both respondents and interviewees were grouped together to find trends in opinion.

We recognised a positive bias could exist. Firstly, the writing of the assessment assumed an impact had occurred. To mitigate this problem, a ‘no influence’ option was included in the questionnaire. Secondly, the respondents that worked on creating the report, or those in close partnership with the writers, may give optimistic estimates of the report’s impact to validate their own work. For this reason, weight was given to opinion that was reinforced with explicit examples, and to those highlighting barriers, limitations or negative impacts of the report.

## Results and discussion

### Key findings

**Figure 2:**
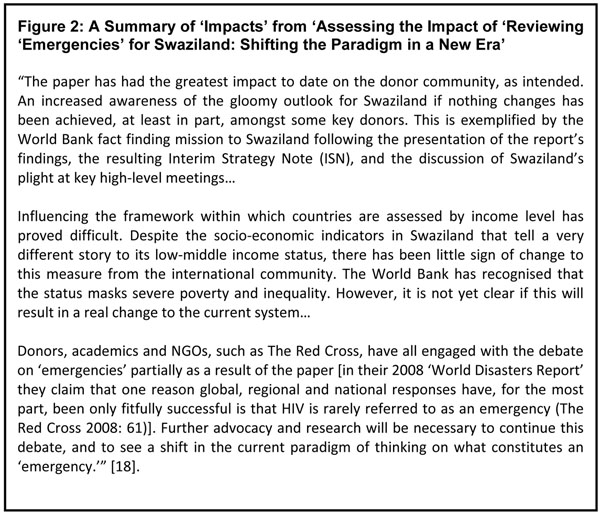
A Summary of ‘Impacts’ from ‘Assessing the Impact of ‘Reviewing ‘Emergencies’ for Swaziland: Shifting the Paradigm in a New Era’

In the assessment the three elements (initial, long-term, and potential) of impact were discussed for each of the five sectors, providing a specific and detailed account of ‘impact’ [13]. See Figure 2 for an overview of these findings. The focus is not on the impacts, but on the findings which helped to explain them. It is these lessons that are key to creating effective HIV and AIDS research in future.

#### The significance of communication

The questionnaire asked, “What is the single most striking aspect of the report (e.g. a graph, a statistic, a statement)?” The graphs were singled out by respondents. Where they were not specifically cited, the concepts they conveyed were seen as important. One respondent answered, “A combination of statistics, graphical illustrations and words are used effectively to convey the message”, illustrating the clear use of these tools in the original report.

**Figure 3:**
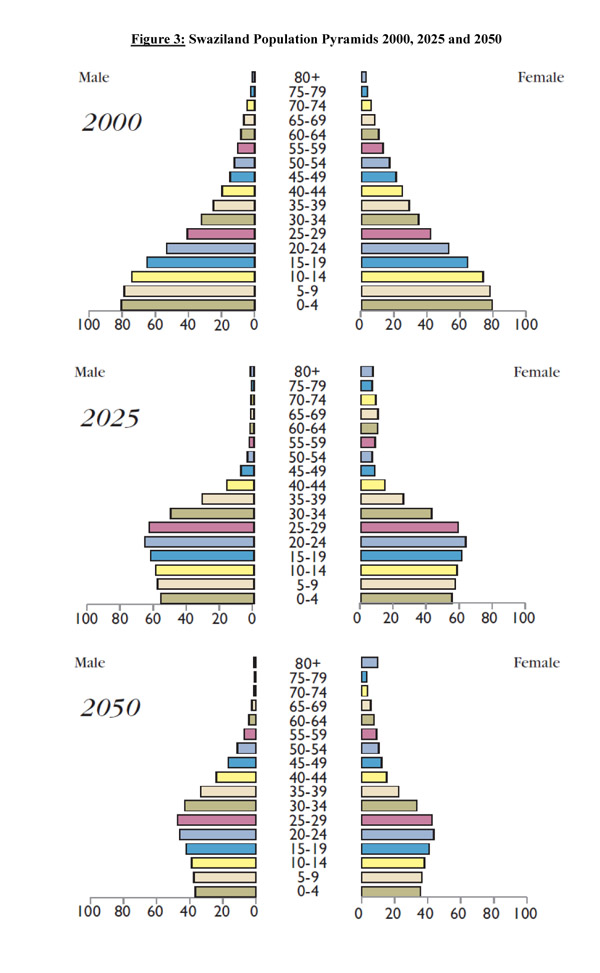
Swaziland Population Pyramids 2000, 2025 and 2050

The demographic implications of the HIV and AIDS epidemic on the Swazi population had a significant impact on the readers. In particular respondents cited ‘Figure 8: Swaziland Population Pyramids’, in Whiteside and Whalley 2007 (shown below) and the concept that a permanent alteration of the structure of Swazi society has occurred. (Figure 3) [14]

The application of Swaziland’s HIV prevalence rate to western countries, in Table 2 (page 8) of the report ‘stuck’ with some. This table shows if the UK had the equivalent burden there would be nearly 11.5 million British citizens infected. When presenting these data, it would be tailored to the audience – for example talking in Sweden the presenter said ‘Swaziland’s prevalence would be equivalent to 1.75 million Swedes being infected’.

It is unsurprising that such dramatic predictions of the effects of the epidemic on the structure of the population would strike the reader. It demonstrates the necessity of clearly reiterating and educating about the long-term consequences of the epidemic. Shortly after the publication of Reviewing Emergencies the government released preliminary data from the national 2007 census showing a decline in the population - 17, 489 fewer Swazis than in 1997 [15]. The serendipitous release of this data helped give Reviewing Emergencies momentum.

Successful dissemination was crucial, but wide dissemination is not the same as wide impact, and it cannot be assumed that the former naturally or inevitably leads to the latter [8]. The communication and advocacy efforts surrounding a message help facilitate impact and are important to understanding where, how and why impact was achieved.

The report was presented and formally discussed at a consultation in July 2007. It was available in print from October 2007, and a core set of power point slides was developed and presented to a range of organisations inside and beyond Swaziland. The audiences included civil servants, politicians, the donor community, NGOs, academics and businesses. Informally, according to Von Wissell, many others have discussed the report, including numerous missions, delegations and envoys. The report was made accessible online by HEARD [5] and NERCHA as well as UNAIDS [16], Relief Web [17], Food, Agriculture and Natural Resources Policy Analysis Network [18] and Aidsportal. (Table 1)

**Table 1:** The Dissemination of “Reviewing ‘Emergencies’ for Swaziland”, July 2007 to April 2008

	***Presentation of ‘Reviewing ‘Emergencies’ for Swaziland’***				
***Month***	**Government**	**Donors**	**NGOs**	**Institutes of Education**	**Business**

**Jul - Sep 2007**	**Drafts circulated for comment to UNAIDS and US Government among others. In August some of the content exposed to NERCHA AGM which is attended by many local stakeholders and donors**				

**Oct 2007**		*SIDA Reference Group, Lusaka*			
	
		**World Bank, including Vice President of the World Bank**			

**Nov 2007**	**Cabinet and Principle Secretaries in Swaziland**	*OCHA/RIASCO, Johannesburg*			

**Dec 2007**	**Parliamentary Portfolio Committees for PM's Office and Health**			*HIV/AIDS and Development. A Case Study from Swaziland AIID Workshop, Amsterdam*	
	
	**Swaziland Partnership Forum on HIV/AIDS**				

**Feb 2008**	**NERCHA Council**	*AIDS and Development: The case of Swaziland’ Rockefeller Brothers Foundation, Cape Town*	**Church Forum, Swaziland**		

**Mar 2008**		**The Donor Forum, Swaziland**	*The AIDS, Development Emergency, Conundrum: A Case Study of Swaziland’ Medicine Sans Frontiers, Brussels*	*The AIDS, Development Emergency, Conundrum: A Case Study of Swaziland’ Institute of Tropical Medicine, Antwerp*	**Royal Swazi Sugar Company and surrounding companies**

**Apr 2008**		*April ‘AIDS impact on economic and other development indicators: the case of Swaziland’ UNAIDS Meeting*	*‘Rethinking Emergencies: Swaziland a Case Study’, Population Council Seminar New York*	*‘Rethinking Emergencies: Swaziland a Case Study’, Harvard School of Public Health Seminar, Cambridge MA USA*	**Federation of Employers and Chamber of Commerce**
	
		**Executive Director of the Global Fund and delegation**	*‘Rethinking Emergencies: Swaziland a Case Study’ IFPRI, Washington*	*‘Rethinking Emergencies: Swaziland a Case Study’ South African Reading Group, New York Law School, New York*	

Presentations in bold were conducted by Dr. Derek Von Wissell, Director of NERCHA, and those in *italics* by Professor Alan Whiteside of HEARD.

HEARD and NERCHA co-ordinated their efforts in communicating the findings. The delivery of the message itself was particularly significant. It had a coherence achieved by using the same slide set – presenters were ‘singing from the same song sheet.’ However, presentations were tailored to the audiences; Von Wissell presented to Swazi audiences in SiSwati; in Scandinavia the importance of donors was stressed.

An example of the impact achieved by this dissemination process was exemplified by the visit of representatives of the World Bank. In November 2007, the Human Development Vice President and Country Director from the World Bank visited Swaziland to assess first-hand the country’s situation in human development and HIV and AIDS. They met with key government officials including the Minister of Finance, and Von Wissell. It was here that Von Wissell presented Reviewing Emergencies. One World Bank official described the effect of this by saying, “It was NERCHA’s presentation that brought home the gravity of the HIV/AIDS situation in Swaziland”, and subsequently the World Bank sent a mission to Swaziland to explore next steps for the Bank. The presentation of the report’s findings was critical to this decision.

#### Engagement, timing and credibility

**Figure 4:**
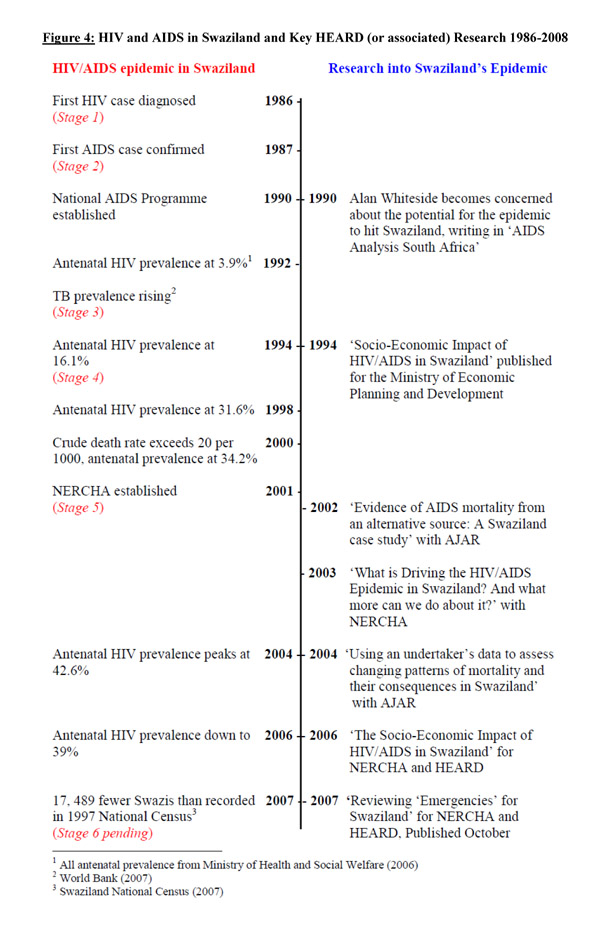
HIV and AIDS in Swaziland and Key HEARD (or associated) Research 1986-2008

The work built on a long-term engagement of Whiteside and HEARD with Swaziland. This historical background is particularly important to both locate the research and the response to it. The timeline below shows the progression of HIV/AIDS in Swaziland through significant events and statistics, simultaneous to key examples of research conducted on the epidemic by HEARD and associates. As the timeline shows, Reviewing Emergencies built on three major reports in 1994, 2003 and 2006, which illustrated there was something going seriously wrong. Its message was credible - based on a history of research and evidence. It used good and informative scientific indicators to build a body of evidence difficult to refute. Furthermore, it was it was written at a critical time in Swaziland: with unusual levels of death; increasing numbers of orphans; and tuberculosis emerging as a major killer, the grim predictions of previous publications were no longer speculative, but reality [5]. (Figure 4)

The timeline illustrates the progression of HIV/AIDS from Stage 1 to Stage 5 in less than 20 years using the concept idea of ‘Stages of the HIV/AIDS epidemic’ developed by Barnett and Whiteside (2002) [19]. As one interviewee reflected, “While, in many ways, Swaziland’s response has been admirable and unique, it is clear that the HIV prevention programmes have not worked thus far, and more importantly, that the social and economic implications of the epidemic have not been adequately thought through.” Reviewing Emergencies tried to explain the significance of the latter, hence filling a crucial gap in the research arena.

Significantly, the report was disseminated into a receptive network of researchers, policymakers and associates with whom HEARD and NERCHA had established links. As one respondent commented, “The wide network of individuals, organisations and donors with whom HEARD and NERCHA are affiliated were key to its wide reception”. The effectiveness of historic relationships such as these, built on both individual and institutional credibility cannot be underestimated. For example Whiteside had served on a UN Commission with the Deputy President of the World Bank, and is a Governor of a school in Swaziland; Von Wissell held the position of Minister of Trade and Industry and Minister of Health in previous governments. The message was delivered by people driven to see change and who could speak with authority. Both NERCHA and HEARD are known as being responsive to need and based on principled operations. In addition, the involvement of NERCHA - a Swazi based and Swazi run body - created an ‘ownership’ of the research and a further credibility to its message.

#### Terminology

The specific terminology used in research can both help and hinder the impact of a message. In Reviewing Emergencies the term and concept of a ‘long-wave emergency’ was particularly significant. A media respondent explained the term ‘emergency’ acted as a “hook”, giving journalists an attention-grabbing story, substantiated by genuine and shocking statistics. Titles such as ‘‘Swaziland: Declare HIV/AIDS a “humanitarian emergency”’ [20] and “When is HIV/AIDS a disaster?” [21] exemplify how the term renewed interest in the epidemic in Swaziland.

Conversely however, concerns over the term ‘emergency’ were discussed at length at the Low-Middle-Income Countries meeting hosted by HEARD in February 2008. One respondent explained, “There is some reluctance on the part of donors/NGOs/civil society/government to call HIV/AIDS an ‘emergency’ because there seems to be fear that terming it so will result in short-term funding for a long-term problem. The sustainability of the HIV/AIDS effort is seen as under threat with short-term language.” This meeting concluded there was a need to marry the urgency of the crisis with a long-wave understanding of the future impact of HIV/AIDS on the country, and that ‘emergencies’ may not be the best term to represent this [22]. A key message of the report is the long-wave nature of HIV and its impacts should be included in a new kind of thinking on emergencies. This debate will continue.

### Barriers and tensions

Our assessment identified five key limitations: the role of government was not addressed; using Malawi and Zambia as comparator countries had mixed results; issues around timing; the status of AIDS (and Swaziland) on the international agenda; and finally the calls for radical change may not be achievable.

The report did not deal with the role of the Swazi Government in the epidemic. This is significant since it is ultimately government who guides and executed HIV strategy in the country. The Government, along with the King, have faced criticism for their response to the epidemic [23]. One respondent reminded us, the crisis “cannot be fixed with more funding alone.” Another discussed the structural difficulties in addressing the crisis, including the ‘vertical’ response, exemplified by a separate AIDS response council (NERCHA) which, it was claimed, failed to join up a national response.

The historic and legal relationship between HEARD, NERCHA and the Swazi Government restrained the report’s ability to criticise. Prescribing how Swaziland should respond to the crisis was never the intention of the paper. Whiteside argued that attempting to change the behaviour of the Swazi Government was beyond the scope of what a researcher from outside of the country should attempt: “That is for the people of Swaziland to do.” Working with the government in a ‘strategic alliance’, rather than against is more productive. Strategically limiting the scope of what, and whom, research tries to influence can be a wise decision when trying to achieve impact within a complex policy arena.

The report made comparisons between Swaziland and Zambia and Malawi, in order to demonstrate the scale of the emergency facing Swaziland. Despite the comparison having the desired effect of emphasising the scale of the Swazi’s crisis - some respondents referenced these comparative graphs as a striking and influential tool - it also had unintended consequences. Zambia and Malawi are both countries that, like Swaziland, compete for the attention of donors and development aid. By focusing all attention on Swaziland, the report appeared to belittle the issues faced by both Malawians and Zambians.

‘Time’ is no doubt a barrier to the impact of the report, since research is usually most influential when first published. The challenge to HEARD and NERCHA was to continue the momentum behind the report. One respondent warned that the report “may lack academic credibility if it is not followed up by further research that is able to collect primary data.” The report aimed for both an immediate awareness of Swaziland’s epidemic, and a longer-term discussion on emergencies and low-middle income classifications. It is the latter of these in particular that needs to be worked at or risk a ‘fizzling out’ as time marches on.

A number of significant international barriers threatened the impact of the report and indeed the issue of HIV/AIDS throughout Sub-Saharan Africa. Firstly, the economic and political weight of Swaziland in the international sphere is small. One frustrated interviewee described that “Swaziland is just not on the list”, as it is considered as insignificant by larger countries. Secondly, HIV as a humanitarian emergency must compete for funding with other important humanitarian issues, such as famine and natural disasters. One respondent described a ‘shift’ in global priorities to issues such as climate change, terrorism and the rising price of food. Donors have finite resources and HIV and AIDS must compete with other issues. HIV and AIDS was the global health issue receiving the most attention and funding but this will change, already a gap exists between pledges and funding [3].

Finally, the report called for a change within the framework that international organisations use to identify countries’ level of development - that of income classification. It further calls for a shift in the paradigm of thinking on emergencies. “Effectively what this report says is that it can’t be business as usual”. Changing entrenched ways of thinking is a major task [24].

## Conclusions

‘Reviewing ‘Emergencies’ for Swaziland’ argued that socio-economic indicators show that the Swazi population is experiencing a humanitarian crisis comparable to countries besieged by conflict or struggling in the wake of a severe natural disaster. In the short-term, the report aimed to focus attention on Swaziland and what AIDS was doing to the country. It contributed to raising the profile of the plight of the Swazi people amongst donors and policy makers, described by one interviewee as a “catalyst for re-engagement”. In the medium-term, the report aimed to influence debate on the classification of low-middle income countries. This has proved more difficult. In the long-term, the report has seen some success in opening the debate on HIV/AIDS as a ‘long-wave emergency’. Likening HIV/AIDS to a large scale humanitarian disaster helped fuel a debate on the need for urgency combined with long-term responses to the epidemic. Donors, academics and NGOs have all engaged with the debate, but further advocacy and research is necessary to continue this, and see a shift in the current paradigm of thinking on what constitutes an ‘emergency.’

Developing a better understanding of the relationship between research and policy impact is vital to advancing the influence of SRH and HIV and AIDS research. Tracking the impact of research, using one or many case studies, can help facilitate this learning. Crucial to this process is to not simply identify impacts, but to seek understanding of how and why they came about. Of particular use for developing effective research in the future is identifying where intended impact has not been achieved and why. The assessment of the impact of Reviewing Emergencies attempted to do exactly this.

Drawing from the assessment, the following lessons may help inform future SRH and HIV/AIDS research. Communication is critical. The original work - collecting existing data to tell one clear story - is a striking way to demonstrate the reach and scale of disease impact; the demographic implications of AIDS powerfully communicated the severity of the epidemic; a targeted, tailored and cohesive dissemination effort helped facilitate the impact of research; sustained advocacy which is vital in keeping momentum for a message, should be reflected in planning and resources; further publications validating and extending a message are a good way to do this; and terminology can help or hinder the impact of a message.

Context and timing may be beyond the control of researchers but can significantly influence the uptake of research. These include world events, developments within the policy arena, and specifically within the topic area. If these conditions align in the favour of the research the impact may be much greater, therefore awareness of this, and careful timing of publications and advocacy efforts could maximise impact.

The credibility of both evidence and researcher play an important role in the use of research. Historical integrity of the evidence and an established researcher (or institution) can foster confidence in the use of research and increase the likelihood of it being used to inform policy. Established relationships and networks between individuals and institutions can guarantee an audience and encourage a dialogue on the findings. ‘Ownership’ of research, by the people it affects, is a powerful way to ensure both credibility and drive behind the message.

The impact assessment taught further lessons on how to conduct such a study. An assessment should ideally be planned in advance, facilitating the process of ‘tracking’ and information gathering; careful attention should be paid to the measurement and analysis process – if basing the study on qualitative data, how will this be analysed and how will the effects of bias be mitigated; the ‘net should be cast wide’ when considering areas of potential impact, recognising both the multiplicity of policy players and the potential for unintended impacts; understand that impacts may also change over time; and ultimately, to understand why an impact took place, the socio-cultural, political and economic context must be considered.

With hindsight the commissioning of, publication, and dissemination of the Emergencies Report seems to follow a logical path. The sense of frustration and the need to provide evidence led to this innovative work. It is our belief that it achieved many of its goals and spurred an international dialogue around the issues. The evaluation taught us additional lessons, which can be applied to help maximise the impact of research in the future.

## Competing interests

This article critically reflects on a research project in which the authors have been involved.

## Authors’ contributions

AW conceptualised the paper, and was co-author to the original study entitled ‘Reviewing ‘Emergencies’ for Swaziland’ (2007). AW also edited the manuscript. FH drafted the manuscript, with input from AW. FH was the author of the study ‘Assessing the Impact of ‘Reviewing ‘Emergencies’ for Swaziland: Shifting the Paradigm in a New Era’ (2008). Both authors read and approved the final manuscript.

## Authors information

AW is Director of the health economics and HIV/AIDS Research Division (HEARD) at the University of KwaZulu-Natal. He has an MA from the School of Development Studies at the University of KwaZulu-Natal and a D Econ from the University of Natal.

FH has an MSc Development Management at the Department of International Development from the London School of Economics and MA Economics and Politics from Edinburgh University. She was a visiting fellow to HEARD in 2008.
